# Association between MUC1 rs4072037 polymorphism and *Helicobacter pylori* in patients with gastric cancer 

**DOI:** 10.22088/cjim.15.1.15

**Published:** 2024

**Authors:** Ramin Shekarriz, Hadi Jabbari, Reza Alikhani, Reza Alizadeh-Navaei, Mohammad Bagher Hashemi‑Soteh

**Affiliations:** 1 Department of Hematology and Oncology, Gastrointestinal Cancer Research Center, Mazandaran University of Medical Sciences, Sari, Iran.; 2 Department of Internal Medicine, Mazandaran University of Medical Sciences, Sari, Iran.; 3 Gastrointestinal Cancer Research Center, Mazandaran University of Medical Sciences, Sari, Iran.; 4 Immunogenetics Research Center, Cell and Molecular Research Center, Faculty of Medicine, Mazandaran University of Medical Sciences, Sari, Iran

**Keywords:** Gastric cancer, *H. pylori*, *MUC1* 5640G&gt;A polymorphism, PCR-RFLP, Genotype.

## Abstract

**Background:**

The *MUC1* gene encodes glycoproteins attached to cell membrane that play a protective role in gastric cancer and protect epithelial surfaces against external factors such as* Helicobacter pylori*. *H. pylori *infection can induce a cascade of innate and acquired immune responses in gastric mucosa. Relationship between rs4072037G>A polymorphism of *MUC1* gene and increased susceptibility to *H. pylori *infection aimed to investigate in patients with gastric cancer in Mazandaran, northern Iran.

**Methods:**

A case-control study was conducted on 99 patients with gastric cancer (*H. pylori *positive and negative) and 98 controls (*H. pylori *positive and negative) without gastric cancer (confirmed by pathological biopsy samples obtained during endoscopy). *H. pylori* infection was diagnosed by histological examination using Giemsa staining. Genomic DNA extracted from peripheral blood was analyzed by PCR-RFLP technique.

**Results:**

Analysis of all genetic models showed no significant relationship between rs4072037G>A polymorphism and risk of gastric cancer (GC). The relationship between *H. pylori *infection and rs4072037G>A polymorphism showed an increased susceptibility to gastric cancer in both positive and negative *H. pylori *groups (including case and control groups). The genetic model of GA/GG and *H. pylori*- positive versus GA/GG and *H. pylori*-negative showed a significantly increased susceptibility to gastric cancer (OR=0.251, CI: 0.128-0.493, P=0.000).

**Conclusion:**

These findings indicate that rs4072037G>A polymorphism may interact with *H. pylori *infection to increase the risk of GC.

Cardiac and non-cardiac gastric cancers are common all over the world. Gastric cancer with more than 1,000,000 new cases and 783,000 deaths (about 1 in 12 deaths worldwide) is the fifth most common cancer in the world and was the third leading cause of cancer death in 2018 (1). The prevalence of gastric cancer is more than twice higher in men than women. Gastric cancer is the most common cancer in men and the leading cause of death in several West Asian countries such as Iran, Turkmenistan, and Kyrgyzstan (1). The prevalence rate of gastric cancer in Iran is reported to be as high as 7300 cases per year and is the most common cancer among both sexes (2). Gastric cancer is highly prevalent in northern provinces of Iran, including Mazandaran, Golestan, and Ardabil (3, 4). Involvement of different environmental factors such as alcohol, smoking, diet (high salt intake and low fruit consumption), and infectious agents like *H. pylori *has been investigated in gastric cancer (1). 

The prevalence of *H. pylori* infection varies in different countries. *H. pylori *contamination is prevalent in about 70% of Iranian people (5) and is responsible for 90% of new cases of noncardiac gastric cancer (1). *H. pylori* is a gram-negative, microerophilic and flagellate bacterium (6). Most people are exposed to *H.pylori* infection during childhood and usually develop gastritis during lifetime. The bacterium causes chronic inflammation and significantly increases the risk of developing gastric ulcer disease and gastric cancer which is the result of *H.pylori* binding to gastric mucosal epithelial cells, inducing an inflammatory response, gastric epithelial cells mutation, inhibition of apoptosis, stimulation of angiogenesis, and cell proliferation (7, 8). 

*MUC1* gene encodes a membrane-bound glycoprotein that binds to upper surface of the gastrointestinal epithelium by a transmembrane domain and protects epithelial surfaces against harmful environmental factors (9, 10). There are three types of mucin, two secretories, MUC5AC and MUC6, and a membrane bound, MUC1, which is dominant in gastric mucus (11, 12). MUC1 is known as a tumor-associated molecule in many cancers due to overexpression and abnormal glycolysis (13). This gene is involved in transformation of malignancy by interacting or regulating the function of other protein like beta-catenin and p53 (14, 15). 

*MUC1* rs4072037 polymorphism (ref SNP ID: 5640; G> A) is located in exon 2 of the *MUC1* gene. This polymorphism is involved in alternative splicing which can produce two different products: a full-length transcription or an incomplete transcription product without a tandem repeat or TR domain. The conversion of variant A (ACGG) to variant B (ACAG) leads to creation of a new splicing acceptor position. Allele G and A rs4072037 polymorphism lead to long and short *VNTR*, respectively. The rs4072037A allele causes deletion of 9 amino acids in *MUC1* gene product. It is believed that, as a result, this truncated non-functional protein increases the risk of gastric cancer (16-20).

The length of *VNTR* allele of the *MUC1* gene affects susceptibility to *H. pylori *(21). It is reported that the smallest size of *VNTR* allele is associated with *H. pylori* infection (13) which then alters the structure and function of gastric epithelial mucins. MUC1 and MUC5AC are major known mucins that play a protective role against *H.pylori* in carcinogenic process (22). The bacterial gastric pathogen H. pylori expresses several adhesins that target mucin glycan structures. The BabA adhesin binds to the fucosylated ABO/Lewis b antigen (Leb) and H type 1 antigens on mucins, while the SabA adhesin binds to the sialyl-Lewis x (sLex) and sialyl-Lewis an antigens (sLea). The released MUC1 binds to H. pylori and thus can act as a decoy receptor to prevent adhesion of the organism to the epithelial surface. Additional evidence of the barrier function of MUC1 is that knockdown of MUC1 expression results in more H. pylori adhesion even when bacteria lack the BabA and SabA adhesins. MUC1 prevents this type of H. pylori binding likely by steric hindrance due to its long rigid filamentous structure (23).

Association between rs4072037 G>A polymorphism of *MUC1* gene and susceptibility to *H. pylori* in patients with gastric cancer (*H.pylori* positive or negative) compared with healthy individuals (*H. pylori* positive or negative was aimed to investigate in this study.

## Methods

**Population study:** 99 patients were recruited with gastric cancer (78 males and 19 females, mean age: 66.11 ± 9.1 years) from outpatient chemotherapy and oncology wards in Comprehensive Cancer Center in Sari Imam Khomeini Hospital, Iran. The patients were selected based on clinical signs, tumor location, and tumor grade and stage according to WHO criteria. Also, 98 control samples (78 men and 19 women, mean age: 56.95±18.4 years) were selected from patients in Sari Imam Khomeini Hospital due to a gastrointestinal problems. Although a bigger sample size will be better, unfortunately for some limitations, it was not possible to gather more samples in this research. 

Pathological examination of endoscopic biopsy specimens of these patients showed no signs of gastric cancer. The presence of *H.pylori* in gastric biopsy specimens was determined using Giemsa staining. This research was approved by the Ethics Committee of Mazandaran University of Medical Sciences (IR.MAZUMS.IMAMHOSPITAL. REC.1398.6561). Sampling from patients and controls were applied after filling an informed consent form, which is taken from all participants in this research.

**DNA extraction:** Peripheral venous blood (5 ml) was collected in a tube containing EDTA and stored at minus 80 °C. Blood samples were taken from patients before chemotherapy. Also, blood samples were taken from the control group after endoscopy of patients with gastrointestinal symptoms and confirmation of no cancerous mass by a gastroenterologist. Control group with malignant biopsy specimens who were confirmed by pathology examination were excluded from the study. Genomic DNA was extracted using a blood DNA extraction kit (Denazist, Iran) according to the manufacturer's instructions.

**Genotype analysis:** PCR-RFLP method was used to analyze the polymorphism of rs4072037G>A *MUC1* gene. A fragment of 188 bp PCR containing polymorphism rs4072037G>A of *MUC1* gene with a total reaction volume of 25 μl was amplified using two primers: The primers for PCR were designed by ourselves and the fragment sizes also were defined using different bioinformatics softwares, like Primer-BLAST and Gene Runner. Forward primer: TAAAGACCCAACCCTATGACT and Reverse primer: AGAGTACGCTGCTGGTCATACTC. A reaction mixture containing 2 μl of template DNA, 12 μl of 2x PCR Master Mix RED (Amplicon, Denmark), 10 μl of distilled water, and 0.3 μl of both forward and reverse primers. PCR reactions were performed under the following conditions: 94°C for 5 minutes followed by 35 cycles of 94°C for 60 seconds and 72°C for 60 seconds. The annealing temperature was set at 60°C for 60 seconds and PCR product was observed on 1% agarose gel containing Green Viewer ™ DNA dye. The 188 bp PCR products were digested using *ALWNI* restriction enzyme for 5 hours at 37°C according to the conditions mentioned in the enzyme protocol (Thermo-Fisher Scientific, USA). After enzymatic digestion, the products were observed by 2% agarose gel electrophoresis containing Green Viewer ™ DNA dye. Enzymatic digestion products included: undigested homozygous GG genotype (188 bp), digested AA homozygous genotype (114 and 74 bp), and heterozygous GA genotype produced three fragments of 188, 114 and 74 bp (fig.1). After actual restriction enzyme digestion, the PCR fragments were exactly the same size as we designed and predicted.

**Statistical analysis: **Statistical analysis was performed using SPSS V22 software package, (SPSS, Chicago, IL, USA). Chisquare test was used to evaluate the differences in distributions of demographic characteristics between gastric cancer patients and normal controls. The expected frequency of control genotypes was tested against Hardy–Weinberg equilibrium. The odds ratio (OR) and 95% confidence interval (CI) were calculated using a logistic regression model and *p<*0.05 was considered statistically significant. Otherwise, the pooled ORs and 95% CIs without adjustments were calculated for frequencies of rs4072037 (G>A) alleles and genotypes, respectively. We also used two-stage Mendelian Randomisation (MR) method to investigate individual causal relationships between allele status and helicobacter pylori infection and gastric cancer as dependent variable. Statistical power in Mendelian randomization studies was calculated with STATA 11.

## Results

Demographic and pathological information of 99 case and 98 control group (*H.pylori*-negative and *H.pylori*-positive subgroups) are summarized in tables 1 and 2, respectively. No significant relationship was observed between rs4072037 G>A polymorphism of *MUC1* gene and risk of gastric cancer in all genetic models (table 3). The genotype and allelic frequency of rs4072037 G>A and their relationship with gastric cancer and *H. pylori *risk are summarized in tables 4 and 5. Also, the relationship between rs4072037 G>A polymorphism genotypes and pathological features of gastric tumor are summarized in table 6. Also, the relationship between rs4072037G> A polymorphism genotypes and *H. pylori* in case and control groups was examined against increased susceptibility to gastric cancer. Findings showed no significant relationship between the frequencies of GG, GA, and AA genotypes and negative *H. pylori* and positive *H. pylori *in case and control groups (P= 0.760 and P= 0.785, respectively) (table 4). When the GA/GG genotype of rs4072037 was considered as a reference in negative *H. pylori*, a significant difference was found between GA/GG genotype in positive *H. pylori *group (OR = 0.251, 95% CI: 0.128–0.493, *p *= 0.000) (table 5). Also a Mendelian Normalisation (MR) test between allele status and helicobacter pylori infection and gastric cancer as dependent variable show that rs4072037 had not causally affected (beta=0.276, se=0.564, P=0.626). The statistical power in Mendelian randomization analysis was 0.42 for AA genotype in compared with GA/GG genotypes. 

**Table 1:** Demographic characteristics of the cases and controls groups

**Sex**	**Control**	**Case**	**P-value**
**Male**	71 (72.4)	80 (80.8)	0.181
**Female**	27 (27.6)	19 (19.2)	
**Age**	56.95±18.4	66.11±9.1	0.000

**Table 2:** Tumor characteristics, including tumor site, tumor grade, lymphatic invasion, perineural invasion, tumor stage and tumor type (n=99)

**Characteristics**		**n**	**Percentage**
**Tumor site**	Cardia	16	16.2
	Non-Cardia	83	83.8
**Grade**	1	6	6.1
	2	56	56.6
	3	37	37.4
**Lymphatic invasion**	Present	62	62.6
	Absent	37	37.4
**Perineural invasion**	Present	36	36.4
	Absent	63	63.6
**T (Tumor)**	1	1	1.0
	2	37	37.4
	3	50	50.5
	4	11	11.1
**N (Nodes)**	0	25	25.3
	1	32	32.3
	2	35	35.4
	3	7	7.1
**M (Metastasis) **	0	71	71.7
	1	28	28.3
**Stage**	1	17	17.2
	2	32	32.3
	3	24	24.2
	4	26	26.3
**Tumor type**	Diffuse	59	60.2
	Intestinal	39	39.8

**Table 3:** Comparison of genotype and allele frequencies of *MUC1* gene polymorphisms between gastric cancer patients (n=99) and normal controls (n=98) using chi-square analysis

**SNP** ^a^	**Genotype/Allele**	**Controls(n=98)**	**Cases(n=99)**	**OR** ^b^ ** (95% CI** ^c^ **)**	***P*** **-value**
***MUC1*** **5640G>A (rs4072037)**	AA	21 (21.4)	16 (16.2)	-	-
	GA	43 (43.9)	47 (47.5)	0.697 (0.323-1.507)	0.359
	GG	34 (34.7)	36 (36.4)	0.720 (0.323-1.604)	0.421
	GA+GG	77 (78.6)	83 (83.8)	0.707 (0.344-1.453)	0.345
	GA+AA	64 (65.3)	63 (63.6)	0.930 (0.519-1.667)	0.807
	AA+GG	55 (56.1)	52 (52.5)	0.865 (0.494-1.516)	0.612
	A	85 (43.4)	79 (39.9)	-	-
	G	111 (56.6)	119 (60.1)	0.867 (0.581-1.294)	0.485

**Table 4:** Interaction of polymorphism genotypes rs4072037G> A in case and control groups with negative and positive *H. pylori* infection

***H. pylori *** **status**	**Genotypes**	**Controls(n =98)**		**Cases(n = 99)**		***P-*** **value**
		n	%	n	%	
**-**	GG	27	36.5	19	43.2	0.760
	GA	31	41.9	17	38.6	
	AA	16	21.6	8	18.2	
**+**	GG	7	29.2	17	30.9	0.785
	GA	12	50.0	30	54.5	
	AA	5	20.8	8	14.5	
**Total**	GG	34	34.7	36	36.4	0.636
	GA	43	43.9	47	47.5	
	AA	21	21.4	16	16.2	

**Table 5:** Joint effects of *H. pylori *seropositivity and variants of the rs4072037 polymorphism on risk of GC

***H. pylori *** **status**	**Genotypes**	**Controls(n =98)**		**Cases(n = 99)**		**OR(95% CI)**	***P-*** **value**
		n	%	n	%		
**-**	GA+GG	58	59.1	36	36.3	-	-
	AA	16	16.4	8	8.1	1.241 (0.482-3.194)	0.654
**+**	GA+GG	19	19.4	47	47.5	0.251 (0.128-0.493)	0.000
	AA	5	5.1	8	8.1	0.388 (0.118-1.278)	0.120

**Table 6:** Distribution of genotypes *MUC1 *rs4072037G>A by tumor characteristics

**Variable**		**rs4072037**							
		**GG**	**GA**	**AA**	***p*** **-value**	**GG+GA**	***p*** **-value**	**AA+GA**	***P*** **-value**
**Tumor site**	Cardia	6 (37.5)	7 (43.8)	3 (18.8)	0.932	13 (81.2)	0.719	10(62.5)	1.000
	Non-Cardia	30 (36.1)	40 (48.2)	13 (15.7)		70 (84.3)		53(63.9)	
**Grade**	I	1 (16.7)	4 (66.7)	1 (16.7)	0.376	5 (83.3)	0.232	5(83.3)	0.386
	II	19 (33.9)	25 (44.6)	12 (21.4)		44 (78.6)		37(66.1)	
	III	16 (43.2)	18 (48.6)	3 (8.1)		34 (91.9)		21(56.8)	
**Lymphatic invasion**	Present	25 (40.3)	28 (45.2)	9 (14.5)	0.554	53 (85.5)	0.583	37(59.7)	0.388
	Absent	11 (29.7)	19 (51.4)	7 (18.9)		30 (81.1)		26(70.3)	
**Perineural invasion**	Present	11 (30.6)	19 (52.8)	6 (16.7)	0.648	30 (83.3)	1.000	25(69.4)	0.394
	Absent	25 (39.7)	28 (44.4)	10 (15.9)		53 (84.1)		38(60.3)	
**T (Tumor)**	T1	0 (0.0)	0 (0.0)	1 (100.0)	0.413	0 (0.0)	0.144	1(100.0)	0.772
	T2	14 (37.8)	18 (48.6)	5 (123.5)		32 (86.5)		23(62.2)	
	T3	17 (34.0)	25 (50.0)	8 (16.0)		42 (84.0)		33(66.0)	
	T4	5 (45.5)	4 (36.4)	2 (18.2)		9 (81.8)		6(54.5)	
**N (Nodes)**	N0	7 (28.0)	14 (56.0)	4 (16.0)	0.745	21(84.0)	0.519	18(72.0)	0.767
	N1	13 (40.6)	16 (50.0)	3 (9.4)		29(90.6)		19(59.4)	
	N2	13 (37.1)	14 (40.0)	8 (22.9)		27(77.1)		22(62.9)	
	N3	3 (42.9)	3 (42.9)	1 (14.3)		6(85.7)		4(57.1)	
**M (Metastasis)**	M0	28 (39.4)	33 (46.5)	10 (14.1)	0.503	61(85.9)	0.377	43(60.6)	0.360
	M1	8 (28.6)	14 (50.0)	6 (21.4)		22(78.6)		20(71.4)	
**Stage**	I	5 (29.4)	9 (52.9)	3 (17.6)	0.588	14(82.4)	0.311	12(70.6)	0.493
	II	14 (43.8)	15 (46.9)	3 (9.4)		29(90.6)		18(56.2)	
	III	10 (41.7)	11 (45.8)	3 (12.5)		21(87.5)		14(58.3)	
	IV	7 (26.9)	12 (46.2)	7 (26.9)		19(73.1)		19(73.1)	
**Tumor type**	Diffuse	19 (32.2)	29 (49.2)	11 (18.6)	0.593	48(81.4)	0.580	40(67.8)	0.396
	Intestinal	16 (41.0)	18 (46.2)	5 (12.8)		34(87.2)		23(59)	

## Discussion

We evaluated the association between polymorphism rs4072037G>A MUC1 gene with Helicobacter pylori in patients with gastric cancer in a case-control study. Previous studies have shown that MUC1 gene polymorphisms are associated with susceptibility to gastric cancer (24, 25). 

Helicobacter pylori causes a wide range of gastrointestinal injuries from gastric ulcers to gastric adenocarcinoma (21). The human mucin (MUC) family consists of MUC1 to MUC21, which are classified into secretory (e.g. MUC2, MUC5AC, MUC5B, and MUC6) and transmembrane members (e.g., MUC1, MUC4, and MUC20) (26, 27)*.* MUC1, MUC4, MUC13, and MUC16 are single pass type I transmembrane proteins and act as a protective mucus gel through their O-glycosylated repetitive ectodomines. MUC1 in the stomach is a ligand for Helicobacter pylori that plays an important role in gastric carcinogenesis (20). The MUC1 N-terminal subunit (MUC1-N) contains highly conserved 20 amino acid tandem repeats (with rs4072037 G allele in *MUC1* gene) that are extensively modified by O-glycans. Alterations in glycosyl-transferase expression in malignant cells leads to glycosylation of MUC1-N tandem repeats (TR domain) (28). 

According to evidence, homozygous AA genotype (small size *VNTR*) is associated with an increased risk of *H. pylori* infection and gastric cancer (29-34). Adherence of H. pylori is dependent on the expression of bacterial adhesins and cognate host glycans, displayed by glycoproteins and glycosphingolipids in gastric epithelium and also by mucins in the gastric mucus layer (35). MUC1 can block the adhesion of *HP *blood group antigen-binding adhesion and sialic acid-binding adhesion to the gastric mucosa, which in turn limits the *HP *colonization, and MUC1 acts as a barrier against exogenous insults in normal epithelial cells. Therefore, low expression of MUC1 may cause a reduction in its barrier function in the stomach and subsequently increases GCa susceptibility (36).

We compared allele (G vs. A) and genotype (GA vs. AA; GG vs. AA) between cases and controls along with dominant model (AG + AA vs. GG), recessive model (AA vs. AG + GG), and over-dominant model (AA+GG vs. AG) (37). Also the association of rs4072037 polymorphism and *H. pylori *with susceptibility to gastric cancer is evaluated. In our previous study which was published earlier, with 91 GC patients and 96 controls, a significant relationship was observed between rs4072037G>A and susceptibility to GC (38). The present study, though did not show such association. Because of the limitation in sample size, which may lead to limited statistical power in this study. Fifty individuals with gastritis were investigated as controls which also may contradict the result if rs4072037G>A play a role in susceptibility to gastric cancer. Similarly, a meta-analysis by Xinyang Liu *et al.* included a hospital-based control group with gastritis and a healthy population-based control group. In that research, hospital-based control group could influence the role of *MUC1* gene rs4072037 polymorphism in carcinogenesis (39). 

Silva *et al*. reported that Colombian and Portuguese patients with chronic gastritis were genetically different in *VNTR* locus. They showed that smaller *VNTR* genotypes of the *MUC1* gene increase susceptibility to intestinal metaplasia in Colombian patients with gastritis. They also observed that *MUC1* gene polymorphism is involved in the development of chronic atrophic gastritis and intestinal metaplasia, which is a precursor to gastric carcinoma (40). Moreover, immunohistochemical analysis of 95 patients with gastritis in Northern Europe showed relation between susceptibility to gastritis and the length of *MUC1* gene functional allele (41)*.*

In the current study, comparison of rs4072037 AG/GG genotypes and H. pylori positive shows an association P= 0.000) with OR =0.251 and 95% confidence interval 0.128-0.493. *H. pylori *causes a wide range of gastrointestinal injuries from gastric ulcers to gastric adenocarcinoma (21). MUC1 membrane mucosa is abnormally expressed in different cancers and in the stomach, it is a ligand for *H. pylori *and plays a key role in gastric carcinogenesis (20). MUC1 is involved in negative regulation of NLRP3 inflammation by regulating NF-κB nuclear factor pathway. Regulation of NLRP3 inflammatory activity by MUC1 is critical for prevention of severe gastritis (28). Variable number of 20 amino acid tandem repeat units (*VNTR *or* TR *domain) lead to polymorphisms in the extracellular domain of MUC1 protein. People infected with *H. pylori *with shorter VNTRs are more susceptible to gastritis and increased risk of stomach cancer (41). MUC1 expression is induced by inflammatory cytokines (tumor necrosis factor-α (TNFα), interferon-γ, IL-6) and Lack of IL-10 and its anti-inflammatory response leads to impaired regulation of MUC1 activation, resulting in chronic inflammation and progression of carcinogenesis. In this regard, the conventional factor-κB kinase-β (IKKβ)–nuclear factor-κB (NF-κB) inhibitory pathway is a possible mediator of cancer progression due to inflammation induced by microorganisms such as Helicobacter pylori (41). Functional polymorphism rs4072037 in *MUC1* gene involved in inflammatory response in patients with *H. pylori* is significantly associated with a predisposition to gastric cancer (32). An immunohistochemical analysis based on TR antibody showed that *H. pylori* may react with MUC1 TR domain on the surface of gastric epithelium and stimulate shedding and internalization. Also, an association was found between functional allele length differences in response to *H. pylori* (41). The association between small sizes of *VNTR* and increased risk of *H. pylori* infection is confirmed (42) and homozygous Short-Short (SS) *VNTR* of the *MUC1* gene is reported to be associated with *H. pylori* compared to the Long-Long (LL) genotype (43, 44). 

A meta-analysis by Beom Su Kim *et al.* 2020, also showed an association between A allele of rs4072037 and GC. For diffuse-type GC, an association was observed for heterozygous GA (GA vs AA), and AG/GG vs. AA in a dominant model. But, no associations were observed with intestinal type-GC (31).

There are some limitations in the present study. First, although age, sex, and tumor site were taken into consideration for subgroup analysis, other important risk factors, such as diet, smoking and drinking status were missing, which might also contribute to the etiology of GC. Second, hospital-based control group limited the role of rs4072037 polymorphism of MUC1 gene in carcinogenesis, so it is also important and may have a different genetic basis in the etiology. Third, the sample size of the cases and control was largely reduced in the analysis, which may lead to limited statistical power in analysis. In the previous study (38), it is not exactly clear that a strong relationship exists between MUC1 rs4072037 polymorphism and gastric cancer. Also, there was not any study in Iranian population available in this regard. More importantly, the relationship between this polymorphism, H. Pylori and gastric cancer is not clear yet. 

The lack of association between rs4072037G> A polymorphism and gastric cancer in this research may be related to small sample size as well as due to the presence of gastritis patients among control group. Also, an association between rs4072037G>A, *H. pylori *and susceptibility to gastric cancer may show an example of gene-environment interactions in etiology of gastric cancer. Future works with larger sample size are needed to be investigate the relation of rs4072037 G>A and risk of gastric cancer in Iranian populations and other ethnicities to prove current findings.
